# Assessing the influence of distinct culture media on human pre-implantation development using single-embryo transcriptomics

**DOI:** 10.3389/fcell.2023.1155634

**Published:** 2023-06-26

**Authors:** Bastien Ducreux, Julie Barberet, Magali Guilleman, Raquel Pérez-Palacios, Aurélie Teissandier, Déborah Bourc’his, Patricia Fauque

**Affiliations:** ^1^ Université Bourgogne Franche-Comté—Equipe Génétique des Anomalies du Développement (GAD), INSERM UMR1231, Dijon, France; ^2^ CHU Dijon Bourgogne, Laboratoire de Biologie de la Reproduction—CECOS, Dijon, France; ^3^ Departamento de Anatomía, Embriología y Genética Animal, Facultad de Veterinaria, Universidad de Zaragoza, Zaragoza, Spain; ^4^ Institut Curie, CNRS, INSERM, PSL University, Paris, France

**Keywords:** assisted reproductive technologies, culture media, embryo, RNA-seq, transcriptome

## Abstract

The use of assisted reproductive technologies is consistently rising across the world. However, making an informed choice on which embryo culture medium should be preferred to ensure satisfactory pregnancy rates and the health of future children critically lacks scientific background. In particular, embryos within their first days of development are highly sensitive to their micro-environment, and it is unknown how their transcriptome adapts to different embryo culture compositions. Here, we determined the impact of culture media composition on gene expression in human pre-implantation embryos. By employing single-embryo RNA-sequencing after 2 or 5 days of the post-fertilization culture in different commercially available media (Ferticult, Global, and SSM), we revealed medium-specific differences in gene expression changes. Embryos cultured pre-compaction until day 2 in Ferticult or Global media notably displayed 266 differentially expressed genes, which were related to essential developmental pathways. Herein, 19 of them could have a key role in early development, based on their previously described dynamic expression changes across development. When embryos were cultured after day 2 in the same media considered more suitable because of its amino acid enrichment, 18 differentially expressed genes thought to be involved in the transition from early to later embryonic stages were identified. Overall, the differences were reduced at the blastocyst stage, highlighting the ability of embryos conceived in a suboptimal *in vitro* culture medium to mitigate the transcriptomic profile acquired under different pre-compaction environments.

## 1 Introduction

Assisted reproductive technologies (ARTs) have allowed the birth of millions of children worldwide. In Europe, for instance, over two million children were born following ARTs (Wyns et al., 2021), and numbers continuously rise, proving that tackling infertility is a huge challenge for decades to come (de Geyter et al., 2020). However, significant variability in ART practice and effectiveness exists between countries and even at the regional scale (Munné et al., 2017; Chambers et al., 2021). In particular, embryo culture is at the core of ARTs, but making an informed decision on which culture medium to use is still a subtle task (Lane and Gardner, 2007). Embryo culture media are not expected to perfectly mirror *in vivo* environment conditions (Vajta et al., 2010), but they should, nonetheless, provide the required biological content to sustain satisfactory embryo development compared to natural conceptions. A myriad of embryo culture media is nowadays commercially available. However, owing to trade confidentiality, their exact composition is unknown, which obscures the scientific decisions for choosing one culture medium over another for embryologists (Biggers and Summers, 2008). Although the competitive commercial race to optimize embryo culture media greatly contributed to increased pregnancy rates in ARTs, the scientific basis behind their formulation is unclear, which is a matter of concern for ART-related biovigilance (Sunde et al., 2016). Very few studies have followed up the health of children born in relation to different embryo culture media used in ART cycles, but they tend to indicate that certain media may be suboptimal, with a potential long-term health impact (Kleijkers et al., 2014; Zandstra et al., 2015; Bouillon et al., 2016).

The early embryo closely interacts with its environment, particularly during the cleavage stage (Zander et al., 2006; Bolnick et al., 2017). After fertilization, the embryo transits through the oviduct until reaching the uterus, where it may be implanted. This journey throughout the maternal track exposes the embryo to multiple molecules, including growth factors, hormones, and metabolites, which promote complex reactions (Paria and Dey, 1990; Kölle et al., 2020; Saint-Dizier et al., 2020). This period also coincides with critical epigenetic reprogramming, which influences gene expression (Morgan et al., 2005; Messerschmidt et al., 2014). Substantial evidence has linked adverse environmental maternal exposures and transcriptome changes in human embryonic stem cells and newborns’ cord blood (Winckelmans et al., 2017; Guo et al., 2019). This likely reflects the adaptation of the embryo to external stressors, displaying remarkable plasticity at the molecular and cellular levels (Bateson et al., 2004; Ramos-Ibeas et al., 2019).

Compared to natural conception, *in vitro* conditions inherent to ARTs can be a source of additional stress (Roseboom, 2018). Indeed, the *in vitro* environment could adversely affect the postnatal phenotype of the offspring born via *in vitro* fertilization (IVF: with or without sperm microinjection) (Fernández-Gonzalez et al., 2004; Watkins and Fleming, 2009; Gardner and Kelley, 2017). The many processes involved in ARTs and, in particular, the osmotic stress, substrate imbalance, volatile organics, and contaminant pollution linked to *in vitro* culture can trigger embryonic stress response mechanisms, but this has been barely assessed to date (Leese, 2002; Puscheck et al., 2015; Cagnone and Sirard, 2016). As Thompson et al. (2007) highlighted, “there is no adaptation by an embryo to its environment that has no consequence.” In particular, differences in embryo culture medium composition may lead to differences in adaptive responses to stress.

The embryo is mostly transcriptionally silent until day 3 (four- to eight-cell stage), when embryonic genome activation (EGA) mainly occurs and relies on maternally provided mRNAs for early embryo development (Braude et al., 1988; Vassena et al., 2011; Leng et al., 2019), although transcription initiation has been reported in human embryos at the one-cell stage, acting as a proxy for early epigenetic programming (Asami et al., 2022). During this period, the capacity of the embryo to maintain metabolism and cellular homeostasis may, thus, be limited (Edwards et al., 1998; Lane and Gardner, 2001). Accordingly, short exposure to ammonium before compaction was shown to compromise the ability to further develop compared with the same exposure after compaction in mice (Zander et al., 2006). After EGA, dynamic changes in gene expression accompany embryonic lineage specification, and anomalies in these sequential expression changes can lead to developmental arrest (Sha et al., 2020). These examples highlight that the early embryo is sensitive to its micro-environment and that many parameters in IVF centers should be tightly controlled, especially embryo culture.

Evidence from animal models showed that *in vitro* culture can affect embryonic gene expression and epigenetic marks compared with *in vivo* conditions (Mann et al., 2004; Rinaudo and Schultz, 2004; Fauque et al., 2007; Wright et al., 2011). Most importantly, these molecular effects can be worsened depending on the culture medium (Rinaudo and Schultz, 2004; Schwarzer et al., 2012; Feuer et al., 2017), with a reported sensitivity of imprinted gene expression (Market-Velker et al., 2010; Ramos-Ibeas et al., 2019). Comparatively, studies in humans have mainly focused on the clinical efficiency of various culture media (live birth rate, implantation rate, clinical pregnancy rate, birthweight, placental weight, and pre-term birth rate) (Dumoulin et al., 2010; Eskild et al., 2013; Youssef et al., 2015; Kleijkers et al., 2016). Only two studies compared the transcriptomic profile of blastocysts cultured in two different media: using microarrays, they reported misregulation of genes involved in cell cycle, apoptosis, protein degradation, and metabolism, which have the capacity to impair embryo development (Kleijkers et al., 2015; Mantikou et al., 2016). This justifies pursuing efforts to identify the biological origin of embryo culture effects.

In this study, we investigated, for the first time, the impact of different culture media used in IVF centers (Ferticult, Global, and SSM) on the transcriptome of a unique collection of day-2 and -5 human embryos using single-embryo RNA sequencing. Analyzing day-2 embryos (four-cell stage) will provide insight into effects of culture media on maternal-provided transcripts and early embryonic gene activation (epigenetic programming). The importance of analyzing day-5 embryos (blastocyst stage) is that it will increase our understanding of effects on molecular processes after compaction. Embryo culture media rich in amino acids, such as Global, are nowadays preferentially used among IVF centers over media depleted in amino acids, such as Ferticult. The SSM medium evaluated in this study is no longer used due to under-performance in terms of pre-implantation and pregnancy rates (Bouillon et al., 2016). In addition, we tested whether supplementation with methionine, an essential amino acid for embryo development, could modulate the embryonic transcriptome.

We found evidence for medium-specific transcriptomic differences at day 2 (embryos at the four-cell stage), affecting major genes involved in embryonic development. In a second experiment, embryos cultured in two different media until day 2 were cultured until the blastocyst stage (day 5) in the same media considered more suitable because of its amino acid enrichment, and differences tended to reduce, reflecting the possible adaptation of the embryonic transcriptome to the culture medium. Using an expression pseudotime approach based on previously described datasets, altered expression of some genes thought to be involved in the transition from early to later embryonic stages was still identified in these blastocysts depending on their culture media pre-compaction. In addition, supplementing the embryo culture medium with methionine nearly four times the concentration found in culture media did not modify gene expression.

## 2 Materials and methods

### 2.1 Ethics statement

This research was authorized by the National Biomedicine Agency (legal decision published in the Journal Officiel under the reference JORF N°0233- 6 October 2016 and extended under the reference JORF n°0303- 31 December 2019).

### 2.2 Embryo selection- experimental design

**FIGURE 1:**
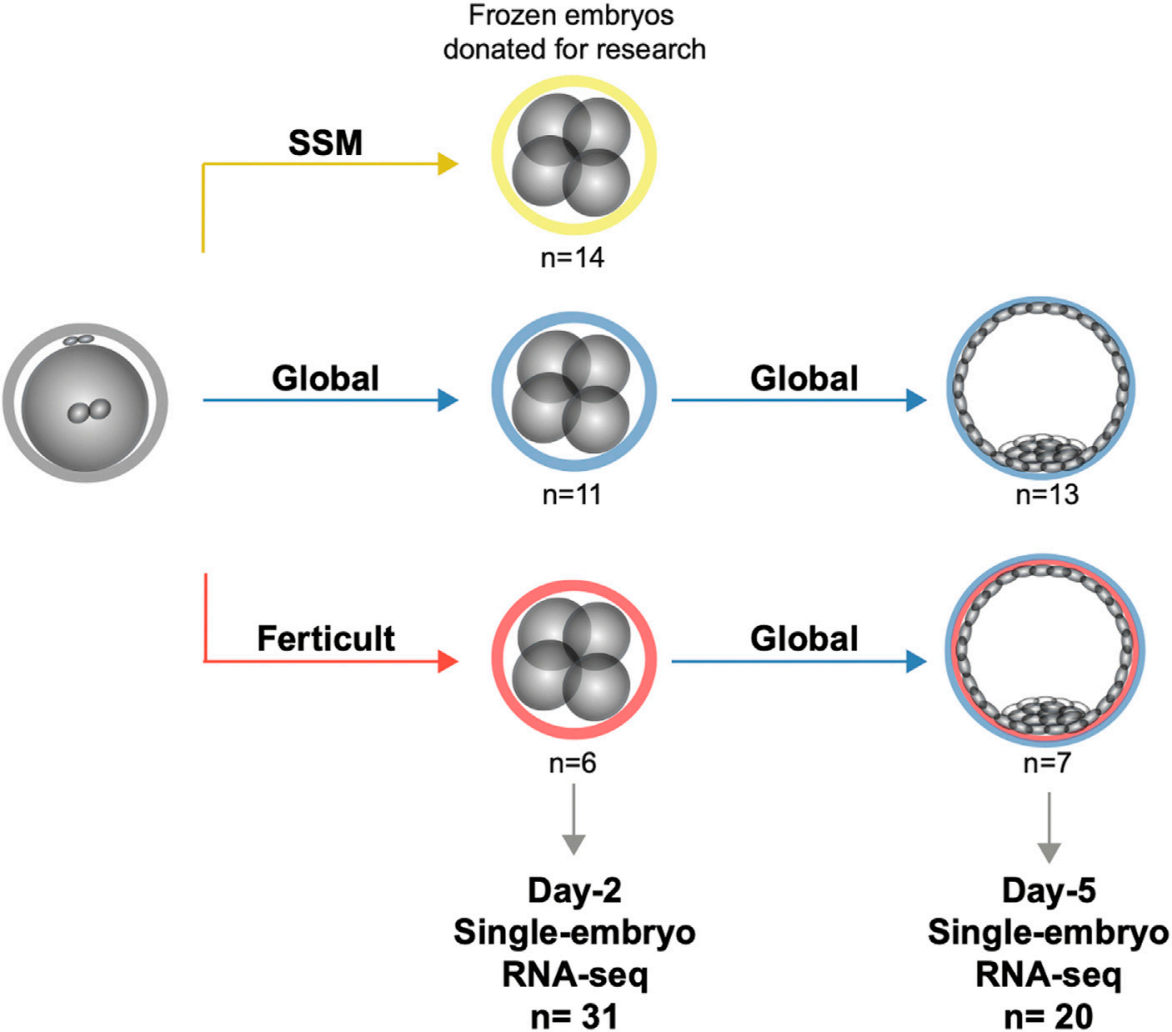
Study design for the transcriptomic analysis of human embryos cultured until days 2 and 5.

We used embryos donated for research by couples and cryopreserved at the Reproductive Lab of Dijon Hospital during a relatively short period (maximum 2 years long). Embryos were included if they originated from couples ≤42 years of age, in conventional IVF or intracytoplasmic sperm injection (ICSI) attempt. Embryos from attempts with surgical spermatozoa (testicular or epididymal sperm) or performed in a viral context (HIV or viral hepatitis) were excluded. Clinical information and the number of embryos per couple are available in Supplementary Table S1. Freezing and thawing were performed with strict procedures as previously described (Bechoua et al., 2009) and detailed in Supplementary Methods. In brief, all embryos used in this study were cryopreserved individually 2 days post-fertilization by a slow-cooling protocol. Then, embryos were *in vitro* cultured in different culture media (Global medium, LifeGlobal; Ferticult IVF medium, FertiPro; SSM, and Irvine Scientific). However, to limit environmental variability, experiments were performed in parallel from different groups, using the same consumables and equipment. We analyzed only embryos cultured in these three different culture media but with identical morphological criteria, i.e., at the four-cell stage with less than 15% of anucleate fragments and regular cleavage (Figure 1). In another experiment, we selected day-2 cryopreserved embryos with identical morphological criteria as described previously from embryo cohorts cultured up to day 2 either in Ferticult or Global, which were then cultured up to day 5 in Global. Finally, for methionine supplementation, we included day-2 cryopreserved embryos from cohorts from the same patients (sibling embryos) with at least four embryos with identical embryo morphological criteria (four-cell stage). Precisely, after thawing, these sibling embryos were randomly cultured in the Global medium without or with methionine supplementation (200 µM; concentration nearly thrice that in the Global medium), and we analyzed by single-embryo RNA-seq sibling embryos that reached the blastocyst stage in both groups (without or with methionine supplementation).

Embryos were thawed according to the strict protocol routinely used in human IVF-clinic to maintain their integrity as much as possible (Bechoua et al., 2009). Immediately after thawing, embryos were transferred into pre-equilibrated embryoslides (Unisense Fertilitech, Vitrolife) with 25 μL of the culture medium and covered with 1.2 mL of oil (Nidoil, Nidacon). They were cultured up to the blastocyst stage at 37°C and tri-gas atmosphere (6%CO2, 5%O2, and 89% N2). According to the classification of Gardner and Schoolcraft (1999), only blastocysts with at least a B2 blastocoel cavity without lysis were analyzed. At the time of sequencing, embryos were between the B2 and B4 blastocyst stages (Supplementary Table S1). We also paid attention to using the same batches of culture media in all experiments.

### 2.3 Single-embryo RNA sequencing

A previously described scRNA-seq method was applied to single embryos (Pérez-Palacios et al., 2021). In brief, zona pellucida-free embryos (after using acidic Tyrode’s solution) were individually placed in a lysis buffer containing 1.35 mM MgCl2 (4379878, Applied Biosystems), 4.5 mM DTT, 0.45% Nonidet P-40 (11332473001, Roche), 0.18 U/mL SUPERase-In (AM2694, Ambion), and 0.36 U/mL RNase-inhibitor (AM2682, Ambion). Then, we performed a reverse transcription reaction (SuperScript III reverse transcriptase—18,080–044, Invitrogen, final concentration: 13.2 U/mL) and poly(A) tailing to the 3’ end of the first-strand cDNA (by using terminal deoxynucleotidyl transferase—10,533–073, Invitrogen, final concentration: 0.75 U/mL). After the second-strand cDNA synthesis, 20 and 18 cycles (at day 2 and day 5, respectively) of PCR were performed to amplify the embryo cDNA using the TaKaRa ExTaq HS (TAKRR006B, Takara, final concentration: 0.05 U/mL) and IS PCR primer (IDT, final concentration: 1 mM). Following purification using a Zymoclean Gel DNA Recovery Kit (ZD4008, Takara), product size distribution and quantity were assessed on a Bioanalyzer using an Agilent 2,100 high-sensitivity DNA assay kit (5,067–4,626, Agilent Technologies).

The library preparation (KAPA Hyper Plus Library prep kit) and sequencing were performed by the ICGex – NGS platform (Institut Curie) on HiSeq 2,500 for day-2 embryos and on NovaSeq 6,000 Illumina sequencer for day-5 embryos for 100 bp paired-end sequencing.

### 2.4 Data pre-processing and quality control

We computed sequencing quality checks with FastQC v0.11.9 and trimming of adapters and low-quality sequences using TrimGalore! V0.6.6. Paired-end read alignment was performed onto human reference genome (hg38) with STAR v2.7.9a (Dobin et al., 2013) reporting randomly one position, allowing 6% of mismatches. Following previous recommendations (Teissandier et al., 2019), repeat annotation was downloaded from RepeatMasker and joined with basic gene annotation from Gencode v19. The merged file was used as an input for quantification with featureCounts v2.0.1. Genes with a minimum of count per million (cpm) > 1 in at least four samples were retained for further analysis. Principal component analyses were implemented with PCAtools v2.8.0 on log2(cpm+1) for all genes for single datasets and common genes for multiple datasets, excepting the 10% genes with the lowest variance.

### 2.5 Differential expression analysis

Differential expression analysis was performed using *edgeR*’s normalization (v3.38.1) combined with *voom* transformation from the *limma* package v3.52.1. *p*-values were computed using *limma* and adjusted with the Benjamini–Hochberg correction for multiple testing. Genes were declared as differentially expressed if FDR<0.1.

### 2.6 Gene Ontology and gene set enrichment analysis

We used Metascape v3.5 to calculate and visualize over-representation of gene ontologies in our list of differentially expressed genes (DEGs) (Zhou et al., 2019). Metascape applies hypergeometric tests and FDR corrections to identify ontology terms that comprise significantly more genes in a given gene list than what would be expected with a random gene list. For each gene list tested, we provided an appropriate background gene list corresponding to all expressed genes in all samples for a given experiment. We selected “Express Analysis” to capture relevant gene annotations from multiple sources (GO, KEGG, Reactome, canonical pathways, and CORUM). The *p*-value cutoff was kept at 0.01.

Gene set enrichment analysis (GSEA) was implemented with the *clusterProfiler* R package (v4.4.1) setting the adjusted *p*-value significance threshold at 0.05. Beforehand, imputed gene lists were pre-ranked by logFC.

### 2.7 Processing of public single-cell RNA-seq datasets in human embryos

We compared our data with three early embryos single-cell RNA-seq studies (Xue et al., 2013; Yan et al., 2013; Petropoulos et al., 2016). Reads alignment and quantification were executed as described previously onto raw reads downloaded from the European Nucleotide Archive (study accessions PRJNA153427, PRJNA189204, and PRJEB11202).

### 2.8 Trajectory inference and pseudotime computing

After pre-processing, read counts data from all 1,529 cells from the work of Petropoulos et al. were pre-clustered and normalized with *scran* and *scater* packages (v1.24.0) after removing lowly expressed genes. Next, *Seurat* (v4.1.1) was used to scale the data and regress variables on total RNA. To identify genes whose expression dynamically changes across early embryonic development, in a continuous manner, independently of the embryo stage, we applied PHATE dimensionality reduction, a recently developed method that has been previously applied to human embryonic stem cells (Moon et al., 2019). We chose PHATE because of its ability to capture heterogeneity and reduce noise better than other dimensionality reduction methods (Moon et al., 2019). As the information geometry relies on diffusion dynamics, PHATE is especially suitable for early development (Moon et al., 2019). PHATE dimension reduction was applied with *phateR* v1.0.7 embedding three dimensions. We then inferred existing lineages and pseudotime using *slingshot* v2.4.0, a method adapted for branching lineage structures in low-dimensional data.

### 2.9 Differential expression along pseudotime

We used *tradeSeq* v1.10.0 (van den Berge et al., 2020) to fit a negative binomial generalized additive model (NB-GAM) for each gene. After examining diagnostic plots of the optimal number of knots (k) according to the Akaike Information Criterion (AIC), k was set to 6 as an optional parameter in the NB-GAM model. We selected DEGs along pseudotime with *associationTest*() function if *p*-value<0.05 and meanLogFC>2. This function relies on Wald tests to assess the null hypothesis that the expression of a gene is constant along pseudotime. DEGs with the culture medium were cross-checked with the list of DEGs along pseudotime according to Petropoulos et al.’s (2016) dataset. For further investigation, a dataset from Yan et al. (2013) was used to visualize these changes in a larger window, from the oocyte to late blastocyst stage.

## 3 Results

### 3.1 Study design and quality control analysis by comparison with previous studies

To analyze the impact of embryo culture media on the embryonic transcriptome, we performed single-embryo RNA-seq (Pérez-Palacios et al., 2021) on 51 frozen/thawed donated embryos after day 2 or day 5 of culture (Figure 1). Clinical characteristics of donors, embryo origin, and morphology are available in Supplementary Table S1, and information regarding survival after thawing can be retrieved from Supplementary Table S2. We compared three different media: Global (LifeGlobal, United States), SSM (Irvine Scientific, United States), and Ferticult (FertiPro, Belgium). Global and SSM have very similar components, except different forms of glutamine and the presence of taurine in SSM (Bouillon et al., 2016), and are intended to be used as one-step media up to day 5/6 of human embryo development (Supplementary Table S3). Ferticult differs from both in that it does not contain amino acids and is fitted to be used up to day 2/3. We processed 31 day-2 embryos (SSM: n = 14, Global: n = 11, and Ferticult: n = 6) and 20 day-5 embryos (Global: n = 13 without (n = 9) or with (n = 4) methionine supplementation and Ferticult: n = 7). At day 5, the number of samples was independent of the rate of embryos that survived the thawing process and reached at least the B2 stage (42.3% and 50.0% in Global and Ferticult groups, respectively). An average of 3.3 million reads per embryo at day 2 and 13.1 million reads at day 5 were generated, with an average mapping rate of 90.1% across all samples (Supplementary Table S4). We were able to detect the expression of 30% and 26% of all RefSeq genes and transposable elements at day 2 and day 5, respectively.

To assess the quality of our generated single-embryo RNA-seq datasets, we relied on previous high-quality studies that performed single-cell RNA-seq (scRNA-seq) in human early embryos. According to the criteria that an expressed gene should have a count per million (cpm) value greater than 1 in at least half of the samples in each embryo stage, we found consistent numbers of 12,056, 10,022, and 11,213 genes being expressed in four-cell stage embryos in the work of Yan et al. (2013), the work of Xue et al. (2013), and our own dataset, respectively (Supplementary Figure S1). In blastocysts, we identified 12,790 expressed genes in our data, compared with 8,204 genes in Yan et al.’s (2013) dataset in which blastocysts were collected a day later, at day 6.

Principal component analysis (PCA) and hierarchical clustering of global gene expression further confirmed the high similarity of our data with those two previous studies: our day-2 embryo samples clustered near four-cell samples, and our day-5 embryos samples clustered beyond morula and before late blastocyst stages (Supplementary Figures S2A,B).

### 3.2 Transcriptomic comparison of day-2 embryos cultured in different media

**FIGURE 2:**
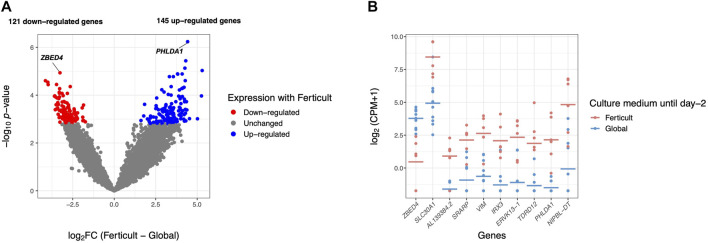
Differential gene expression analysis in day-2 embryos between Ferticult and Global media. **(A)** Volcano plot of gene expression between Ferticult and Global media at day 2. **(B)** Dot plot of the expression of top 10 Ferticult-to-Global DEGs, ordered by ascending log2FC (from left to right). Group mean is represented by the line. Dots represent individual embryos.

We first focused on transcriptome differences in short-term culture, at the four-cell stage (day-2), between embryos conceived in different media. The highest number of DEGs was found comparing Ferticult and Global media groups, with 266 DEGs (1.5% of all transcripts analyzed) showing adjusted *p*-value<0.1 (Figure 2A; Supplementary Table S5). In contrast, only one and five DEGs were found comparing SSM with either Ferticult or Global groups, respectively (Supplementary Table S5). However, the global transcriptomic analysis showed that SSM was transcriptionally closer to Global than to Ferticult (r = 0.95 versus r = 0.9, Spearman’s correlation), which is consistent with their similar composition (Supplementary Figure S3A). Histograms of *p*-value distribution for all genes corroborate the observation of a strong effect of the culture medium on transcriptomic differences between Ferticult and Global and to a lesser extent between Ferticult and SSM, whereas *p*-values tended to be uniformly distributed between SSM and Global and far from statistical significance (Supplementary Figure S3B; Supplementary Table S5).

**FIGURE 3:**
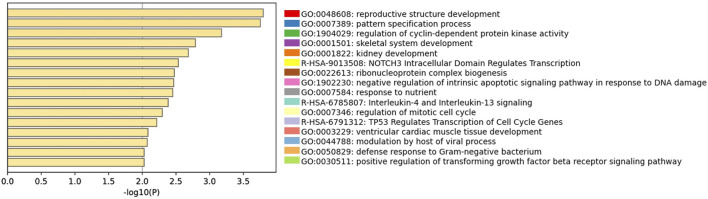
Gene Ontology analyses of Ferticult-to-Global DEGs at day 2. Bar plot of the most significant GO terms from clusters of significant pathways over-represented in day-2 DEGs, ordered by significance. Each term was selected by Metascape using a heuristic algorithm that selected the most informative term from clusters of proximal significant GO terms.

Among the 266 DEGs in the Ferticult-to-Global comparison, 145 were upregulated and 121 downregulated. Most of them (88.3%) displayed absolute log2(fold change, FC) > 2.5, which revealed substantial differences in the transcriptome of day-2 embryos depending on the culture medium (Supplementary Table S5; Supplementary Figures S4, S5A). Only eight DEGs are likely to be maternal transcripts because their expression is strictly declining in embryonic stages succeeding oocyte as assessed with Yan et al.’s (2013) reference dataset (Supplementary Figure S5B). Top 10 most dysregulated genes including *ZBED4*, *SLC30A1*, AL139384.2, *SRARP*, *VIM*, *IRX3*, *ERVK13-1*, *TDRD12*, *PHLDA1*, and *NIPBL-DT* are shown in Figure 2B for each individual embryo according to the culture medium group. Six of them can be considered as mixed maternal/embryonic transcripts, while the four others appear to be transcribed from the embryonic genome. Gene Ontology (GO) analysis with Metascape revealed an over-representation of DEGs related to development (pattern specification process, reproductive structure development, skeletal system development, and kidney development), regulation (regulation of the mitotic cell cycle, regulation of cyclin-dependent protein kinase activity, negative regulation of the intrinsic apoptotic signaling pathway in response to DNA damage, and positive regulation of the transforming growth factor), ribonucleoprotein biogenesis complex, and response to nutrients (Figure 3; Supplementary Figure S6). Adjustments for maternal age or fertilization method were performed in all differential expression analyses, but the top DEGs remained the same (Supplementary Table S5). In parallel, we decided to perform a GSEA which provides a broader view of the overall biological processes that may be up- or downregulated with the use of either Ferticult or Global, which may not be detected by focusing on DEG interpretation. Top 10 significant ontologies indicate that genes involved in embryonic development and cell division are, respectively, likely to be differentially up- and downregulated with Ferticult (Supplementary Figure S7; Supplementary Table S6).

**FIGURE 4:**
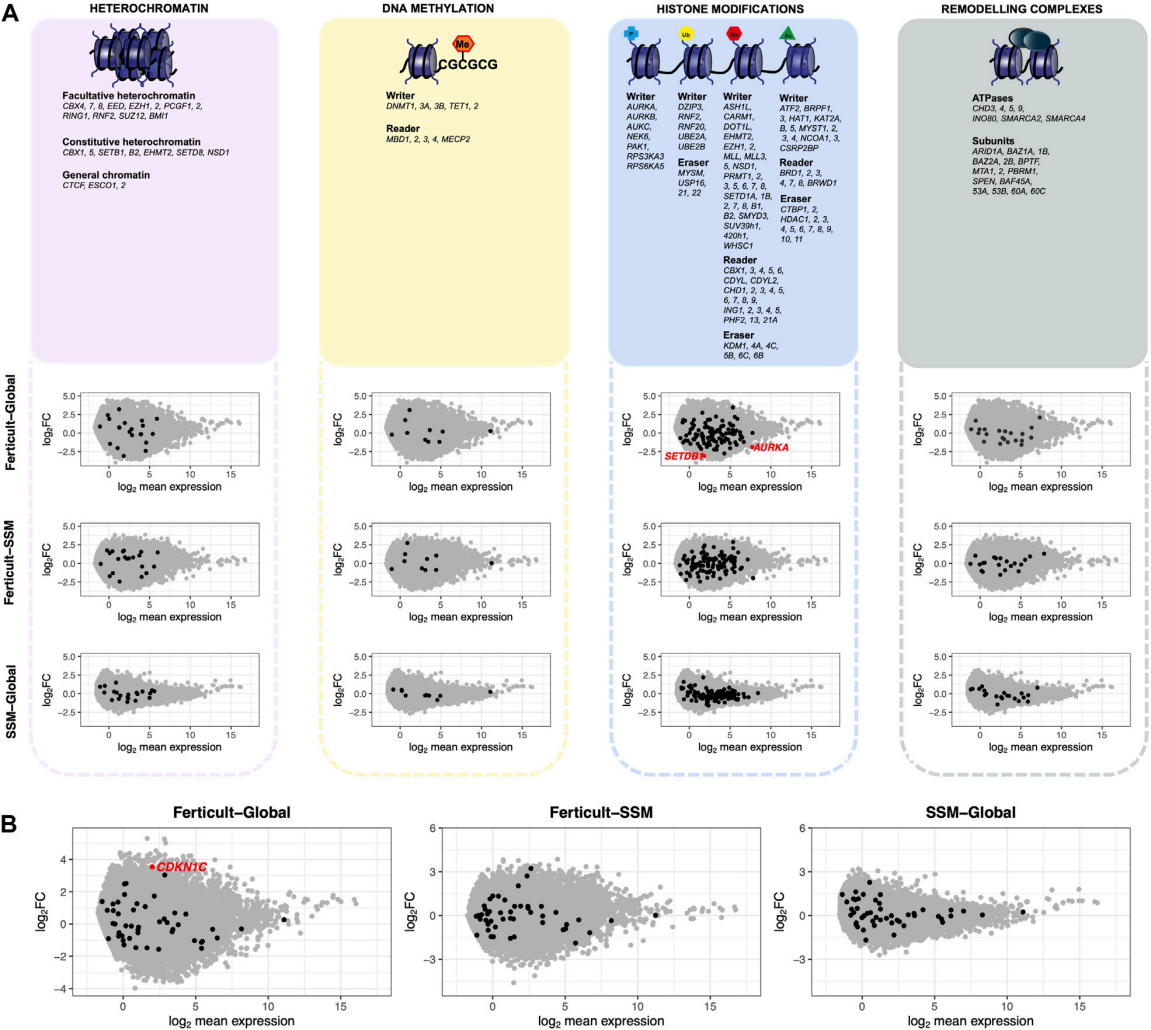
Expression differences of genes involved in chromatin-based mechanisms, imprinted genes, and transposable elements between Ferticult, SSM, and Global samples (day-2 embryos). **(A)** List of major genes identified as involved in chromatin-based processes with their scatter plot of differential expression analysis. Log2 mean expression was calculated by taking the average log2(cpm+1) expression in compared culture media groups. Each point represents a gene: misregulated genes from the aforementioned list (red dots), unchanged genes from the aforementioned list (black dots), and other expressed genes (gray dots). **(B)** Scatter plot of differential expression analysis focused on imprinted genes. Log2 mean expression was calculated by taking the average log2(cpm+1) expression in compared culture media groups. Each point represents a gene: misregulated imprinted genes (red dots), unchanged imprinted genes (black dots), and other expressed genes (gray dots).

Focusing on major genes involved in chromatin-based processes such as DNA methylation, heterochromatin modulators, histone modifiers, and remodeling complexes, we found two histone modifiers to be significantly downregulated among the Ferticult-to-Global DEGs: the Aurora kinase A gene *AURKA* (FDR<0.1, log2FC = −1.84), which regulates many aspects of mitosis, and *SETDB1* (FDR<0.1, log2FC = −2.92), which catalyzes trimethylation of lysine 9 of histone H3 (H3K9me3) (Figure 4A). Additionally, focusing on imprinted genes, we only found the cyclin-dependent kinase inhibitor 1 (*CDKN1C*) gene among the 266 Ferticult-to-Global DEGs (Figure 4B). Finally, we also analyzed transposable elements and found three families to be differentially expressed between Ferticult and Global media (*CR1-12_1Mi*, *LTR6A*, and *LTR7C*). Although the top 20 expressed transposable element families were not differentially expressed in any comparison, their expression was higher in Ferticult, which resulted in fold change intensities higher in Ferticult comparisons (Supplementary Figures S8A,B).

### 3.3 Monitoring 2-day culture medium-induced differences at the blastocyst stage

**FIGURE 5:**
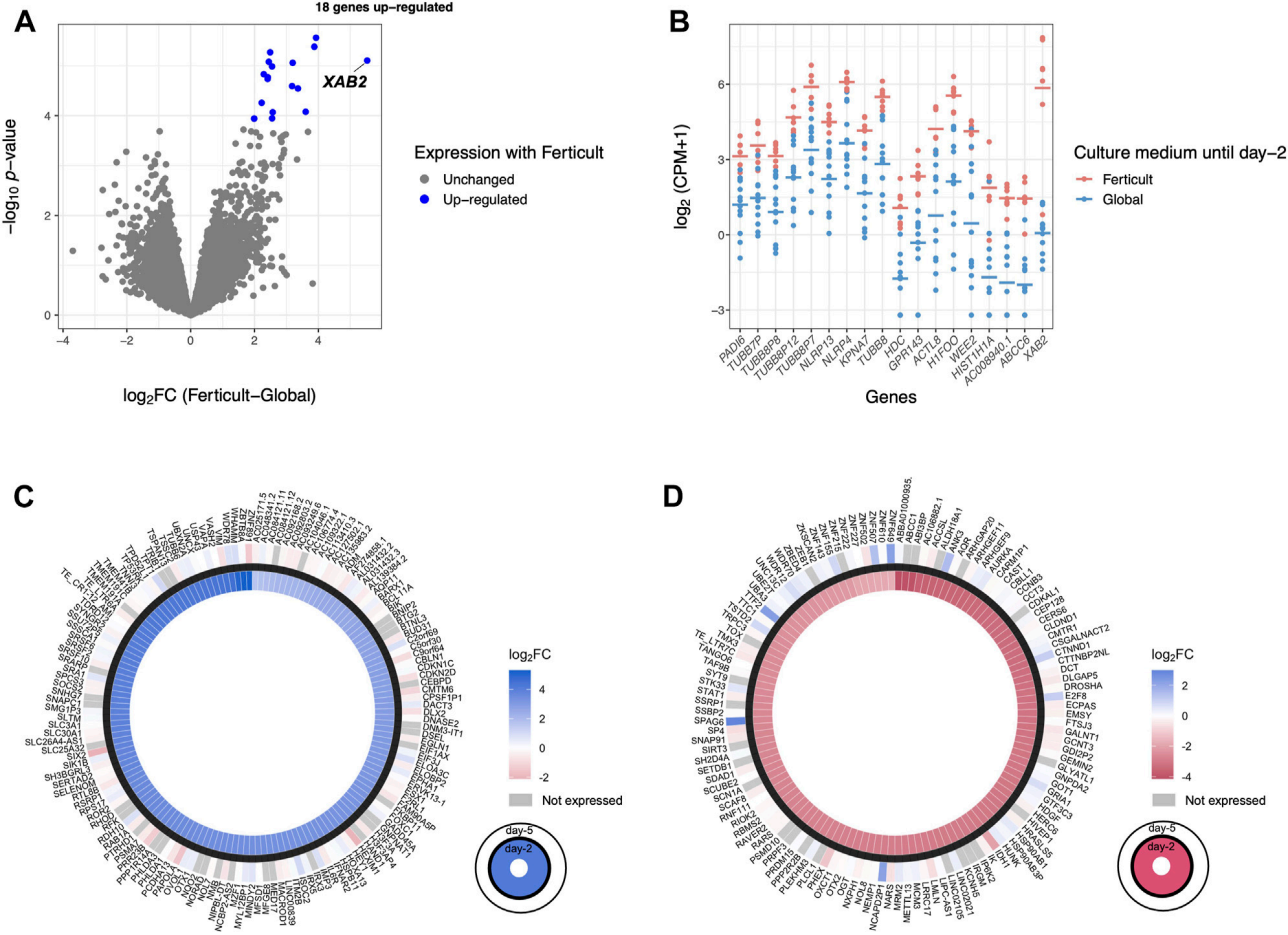
Differential expression analysis at day 5 of culture. **(A)** Volcano plot of differential gene expression between Ferticult and Global media. **(B)** Dot plot of the expression of all 18 Ferticult-to-Global DEGs, ordered by ascending log2FC (from left to right). Group mean is represented by the line. Dots represent individual embryos. **(C)** Circle plot of the expression of upregulated Ferticult-to-Global DEGs at day 2 and their expression at day 5. Plot displays the log2 fold change of the 147 DEGs upregulated with Ferticult at day 2 (interior layer) and the log2 fold change with Ferticult for the same genes at day 5 (exterior layer). Cells colored in gray correspond to genes that were not expressed at day 5. **(D)** Circle plot of the expression of downregulated Ferticult-to-Global DEGs at day 2 Global and their expression at day 5. Plot displays the log2 fold change of the 122 DEGs downregulated with Ferticult at day 2 (interior layer) and the log2 fold change with Ferticult for the same genes at day 5 (exterior layer). Cells colored in gray correspond to genes that were not expressed at day 5.

Given the transcriptomic differences of day-2 embryos resulting from the amino-acid-free Ferticult medium over Global medium, we wanted to further analyze whether amino acid deprivation during the first 2 days of development may have extended effects on the transcriptome of blastocyst embryos. For that purpose, a second batch of embryos cultured until day 2 in Global (n = 13) or Ferticult (n = 7) media was selected for their strict identical embryo morphology and subsequently cultured until the blastocyst stage, all in the Global medium (Figure 1A). Performing differential expression analysis after single-embryo RNA-seq, we found 18 DEGs in blastocysts that were previously cultured in the Ferticult medium until day 2 versus blastocysts cultured all along in Global: *ABCC6*, AC008940.1, *ACTL8*, *GPR143*, *H1FOO*, *HDC*, *HIST1H1A*, *KPNA7*, *NLRP4*, *NLRP13*, *PADI6*, *TUBB7P*, *TUBB8*, *TUBB8P7*, *TUBB8P8*, *TUBB8P12*, *WEE2*, and *XAB2* (Figures 5A,B; Supplementary Table S7). These genes were all upregulated with the Ferticult medium condition until day 2, with half showing a log2FC > 2.5 and the *XAB2* gene showing the highest overexpression score (>5.5) (Figure 5A). The GO analysis indicated a functional link with the meiotic cycle (DEGs associated with this pathway: *H1FOO*, *TUBB8*, and *WEE2*). Importantly, none of the previous expression differences observed at day 2 remained significant at day 5. Circular plots showed that fold changes of gene differences observed at day 2 were largely minimized by day 5 (Figures 5C,D). Conclusions were unchanged when adjusting for maternal age (Supplementary Table S7).

### 3.4 Effects of methionine supplementation

Methionine is an essential amino acid present in embryo culture media that serves as a precursor for protein synthesis and DNA methylation and, therefore, could modulate the transcriptome. We tested whether the addition of methionine at day 2 would impact the transcriptome of day-5 blastocysts cultured in Global by comparing nine samples cultured in Global and four samples cultured in Global supplemented with methionine after day 2, from sibling embryos (i.e., a pair of embryos of each condition were coming from the same couples) (Supplementary Figure S9). The differential expression analysis revealed no significant DEG.

### 3.5 Effects of the culture medium on the transcriptional trajectory of early embryos

**FIGURE 6:**
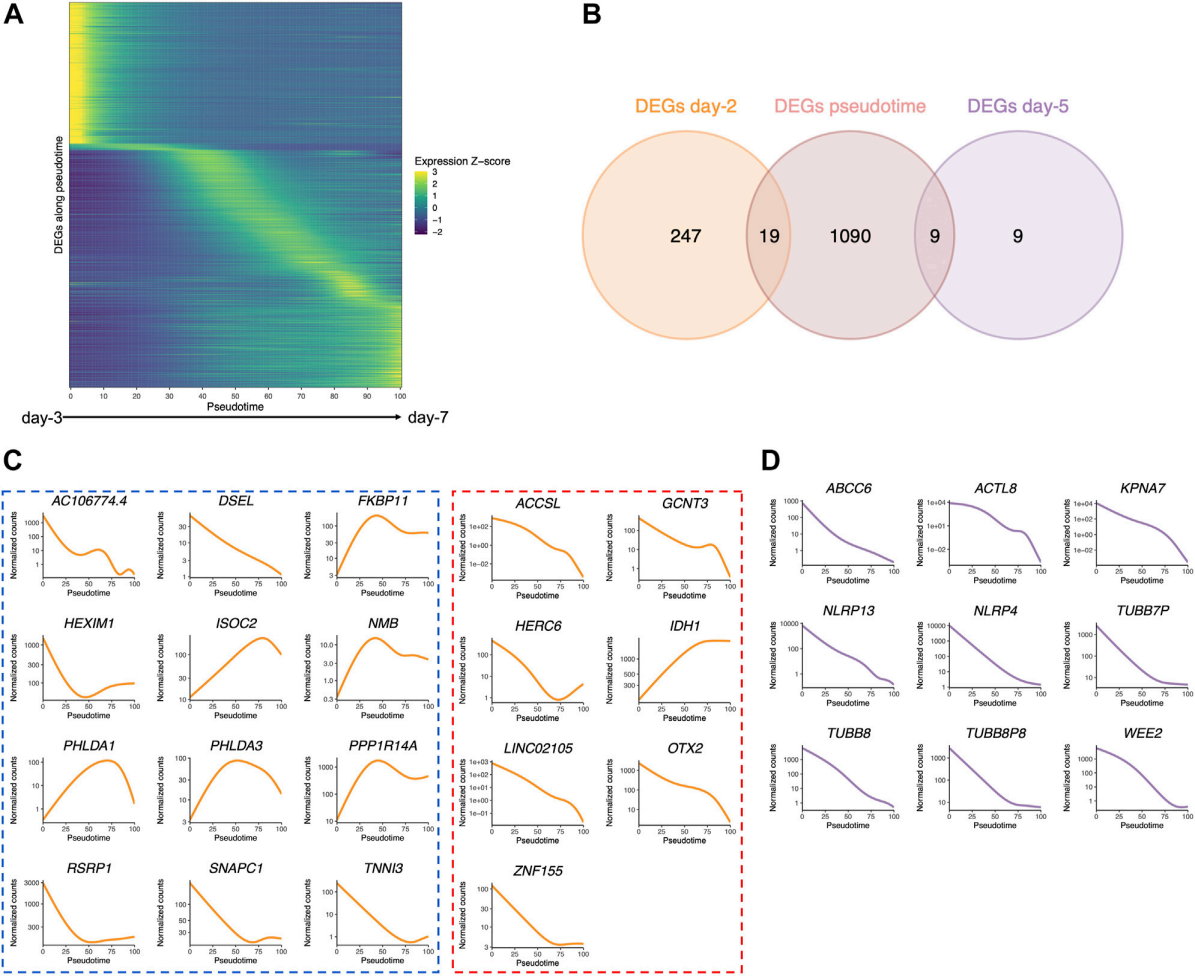
Pseudotime differential expression during human preimplantation development. Pseudotime is a metric that could be interpreted as a timing distance between one cell and its precursor cell and helps identify the ordering of cells along a lineage based on their gene expression profile. **(A)** Heatmap of the expression of the 1,110 genes that were found to be differentially expressed along pseudotime (from public datasets established from the eight-cell stage (Petropoulos et al., 2016), ordered by the timing of peak expression (arbitrary unit). Expression Z-score: Z-score of TMM-adjusted cpm. **(B)** Venn diagram of the number of DEGs along pseudotime that are differentially expressed between Ferticult and Global at days 2 and 5 of the embryonic culture. **(C)** Dynamics of the expression of the 19 day-2 Ferticult-to-Global DEGs that are differentially expressed along pseudotime according to Petropoulos et al.’s (2016) dataset. The curve corresponds to the NB-GAM fitted normalized counts (TMM-adjusted cpm). Left panel surrounded by a blue line corresponds to genes upregulated with Ferticult. Right panel surrounded by a red line corresponds to genes downregulated with Ferticult. **(D)** Expression dynamics of the nine day-5 Ferticult-to-Global DEGs that are differentially expressed along pseudotime according to Petropoulos et al.’s (2016) dataset. The curve corresponds to the NB-GAM fitted normalized counts (TMM-adjusted cpm).

Early embryos undergo profound transcriptional changes during the first stages of development. In an attempt to address whether the embryo culture medium may affect these sequential modifications of gene expression, we used a public dataset of 1,529 single-cell RNA-seq of human embryos from days 3 to 7 (cultured in either CCM (Vitrolife) or G-1 Plus (Vitrolife) media) (Petropoulos et al., 2016), which previously allowed delineating the transcription signature of each embryonic lineage and their dynamics during embryonic lineage segregation (Meistermann et al., 2021). Our objective was to identify genes whose expression dynamically changes across early embryonic development, in a continuous manner, independent of the embryo stage. For that purpose, we applied the PHATE dimensionality reduction method (Moon et al., 2019) and inferred existing lineages and pseudotime (van den Berge et al., 2020)—a metric that could be interpreted as a timing distance between 1 cell and its precursor cell. On the public scRNA-seq dataset (Petropoulos et al., 2016), we were able to identify eight clusters by using k-means clustering to group cells with high transcriptomic similarities. We also identified three distinct lineages (Supplementary Figure S10A) that shared the same structure when considering cells from clusters 1 to 5 but separated into clusters 6, 7, and 8. Using the cell classification adopted in previous studies (Petropoulos et al., 2016; Meistermann et al., 2021), the three lineages corroborated with the demarcation into epiblast (EPI), primitive endoderm (PrE), and trophectoderm (TE) cells (Supplementary Figures S10B,C). Each cell was then assigned a pseudotime to reflect its “transcriptomic age” along each of the three lineages (Supplementary Figure S10D). Along with this inferred pseudotime, we identified 1,110 genes with a dynamic expression pattern (Figure 6A).

**FIGURE 7:**
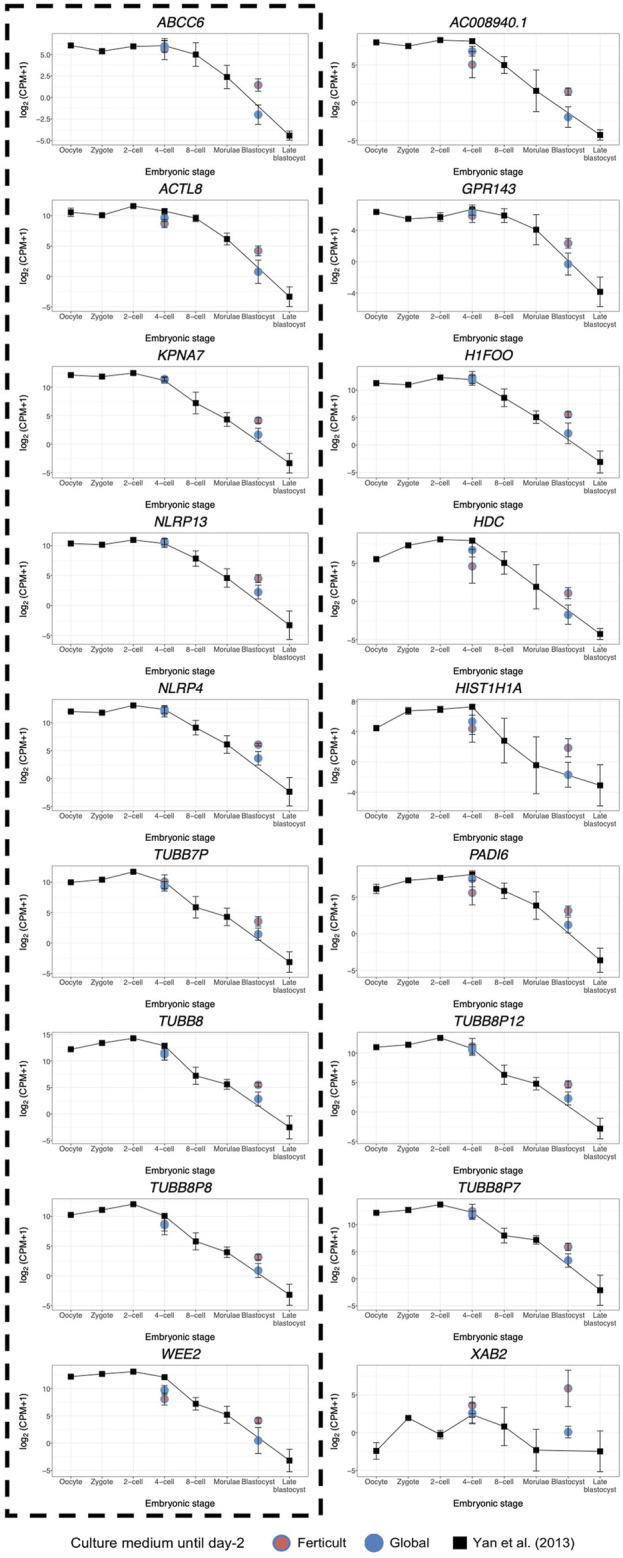
Expression dynamics of day-5 Ferticult-to-Global DEGs from the oocyte to the blastocyst stage as compared to the literature. We plotted the public scRNA-seq dataset from the work of Yan et al. (2013) obtained from the oocyte to the late blastocyst stage as the reference level of the expression of early embryonic genes. Each colored point represents the mean log2(cpm+1) value of all samples for each culture medium group. Black squares represent the mean log2(cpm+1) for all samples from the work of Yan et al. (2013) for each embryonic stage. Error bars represent the standard deviation between samples from the same group. Day-2 embryos were represented at the four-cell stage. Day-5 embryos were represented at the blastocyst stage. DEGs surrounded by a dashed black line were also found to be differentially expressed along pseudotime according to Petropoulos et al.’s (2016) dataset.

Considering the question of the impact of the culture medium, we crossed these 1,110 dynamic genes with our list of DEGs identified at days 2 and 5 in the Ferticult-to-Global comparison. Remarkably, 19 out of 266 DEGs at day 2 and 9 out of 18 DEGs at day 5 showed dynamic expression changes across pre-implantation pseudotime, meaning that the choice of the culture medium has an impact on the expression of genes that are dynamically regulated during early development (Figure 6B). Temporal expression of those genes is shown in Figures 6C,D. While the 19 DEGs at day 2 that were also differentially expressed pseudotime showed a diverse profile of expression (Figure 6C), the 9 DEGs at day 5 that are also differentially expressed along pseudotime displayed a declining expression over embryo development in the reference pseudotime (Figure 6D). To confirm these results, we used an independent public scRNA-seq dataset from the work of Yan et al. (2013) obtained with a broader window, from the oocyte to late blastocyst (Figure 7). When considering our own datasets, embryos continuously cultured in Global up to day 5 also showed this declining trend of expression from days 2 to 5, with levels that were congruent with the reference dataset from the work of Yan et al. (2013) (Figure 7). However, day-5 embryos previously cultured in Ferticult until the four-cell stage showed over-expression for all DEGs, suggesting that these embryos retained abnormally high levels for their embryonic stage. On average, these blastocyst embryos showed expression levels that were closer to the morula stages.

## 4 Discussion

We provide here in-depth characterization of the transcriptomic effects exerted by different culture media on human embryos after 2 and 5 days of culture. In this study, media determined as a worse-case scenario, Ferticult and SSM, were compared to Global, as a better-case scenario, providing insight into the impact of culture media on the transcriptome of early ART-produced human embryos. It should be noted that for the best-case scenario, *in vivo*-derived embryos cannot ethically be obtained. It yields several insights into how culture medium composition can induce transcriptomic responses as an adaptation of the embryo to its micro-environment, pre- and post-compaction. In line with the importance of our research questions, two of the media we tested are no longer used for human embryo culture (one was removed from the market).

First, we focused on embryos cultured until day 2, as this pre-compaction period is likely to be sensitive to environmental stressors. The most pronounced transcriptional divergence was between Ferticult and Global, with 266 DEGs. Among these DEGs, the majority were transcribed from the embryonic genome or consisted of mixed maternal/embryonic transcripts. It is in agreement with studies using animal models, where it has been shown that the culture environment influences the maternal-to-embryonic transition, which itself influences the maternal transcript clearance (Tesfaye et al., 2004; Zhang et al., 2022). A total of 19 of these DEGs could have a key role in early development, based not only on their dynamic expression changes across development but also on their association with GO terms related to essential developmental pathways, and were mostly upregulated in Ferticult. GSEA also depicted global differential regulation of important developmental pathways regarding the use of different culture media, notably cell division and pattern specification processes. Our results are congruent with two previous microarray studies that measured transcriptomic differences between embryos cultured in two culture media (G5 and HTF) related to cell cycle and metabolism-associated genes (Kleijkers et al., 2015; Mantikou et al., 2016). Despite SSM and Global having proximal components, the Ferticult–SSM comparison yielded only one DEG, but the overview of global patterns of expression still assumes the existence of transcriptomic differences between Ferticult and SSM media. The few composition disparities between SSM and Global would explain why the embryonic response to the SSM culture is not completely equal to that of Global.

Imprinted genes are candidates for high susceptibility to environmental conditions, and disruption of imprinted expression has been linked to developmental pathologies in humans (Maher and Reik, 2000). Accordingly, animal studies indicated that some embryo culture media were associated with hypomethylation of maternally expressed genes (such as *H19* and *SNRPN*), resulting in aberrant biallelic expression (Mann et al., 2004; Market-Velker et al., 2012). In our study, only one imprinted gene, *CDKN1C*, was upregulated after 2 days in culture in Ferticult compared to Global. *CDKN1C* is a key regulator of cell growth and proliferation, and aberrant expression is observed in syndromes with overgrowth, tumor predisposition, and congenital malformations, such as Beckwith–Wiedemann syndrome, notably in mouse embryos and fetuses (Andrews et al., 2007; Tunster et al., 2011). We also found that the expression of *SETDB1*, which is involved in histone methylation, was downregulated in Ferticult samples. These elements may reflect direct and indirect influences of the culture medium on the embryonic epigenome.

The link between culture medium composition and transcriptomic effects is still unclear, but transcriptional changes may reflect an adaptation to a sub-optimal environment. For those reasons, we further tested whether the differences observed at day 2 in Ferticult over Global were maintained later on, at the blastocyst stage, after being cultured in Global, which is considered more suitable because of its amino acid enrichment (Rinaudo and Schultz, 2004). Only 18 DEGs were retrieved, and importantly, none of the early differences observed at day 2 were conserved at this later stage. Notably, the differences in expression levels of *AURKA*, *SETDB1*, and the imprinted *CDKN1C* gene observed at day 2 no longer existed at day 5. The original transcriptional changes may not have persisted because, in post-compaction, the embryo acquires an increasing ability to mitigate the transcriptomic profile acquired under different pre-compaction environments and to correct transcriptional errors (Wale and Gardner, 2016). A second hypothesis is that the Global medium composition itself may have allowed the embryo to recover a favorable transcriptomic landscape. Finally, we cannot rule out that only viable embryos were able to develop until the blastocyst stage, and only embryos with functional abilities were, therefore, selected in our analysis.

Our analysis of genes that are differentially expressed along pseudotime brought evidence that the use of distinct culture media prior to compaction can alter the sequential gene expression changes linked to later embryo development. Genes activated or downregulated at the wrong time may impact development and cause lasting effects (Calle et al., 2012; Bertoldo et al., 2015). Notably, Ferticult was associated with the over-expression of some genes at day 5. It is, therefore, possible that 2-day culture in Ferticult induces a delay in clearance of some maternal RNAs. Accordingly, two of the 18 DEGs at day 5 were maternal effect genes (*PADI6* and *TUBB8*) (Mitchell, 2022), which may indeed reflect longer retention of maternal transcripts. *PADI6* is a member of the subcortical oocyte complex (Yu et al., 2014; Bebbere et al., 2016), while *TUBB8* is the major constituent of the oocyte meiotic spindle assembly in primates (Feng et al., 2016).

Nevertheless, our pseudotime analysis of genes differentially expressed over the course of development also identified genes that are thought to be involved in the transition from early to later embryonic stages, such as *ABCC6*, *ACTL8*, *KPNA7*, *NLRP4*, *NLRP13*, *TUBB7P*, *TUBB8P8*, and *WEE2*. *TUBB7P* and *TUBB8P8* encode beta-tubulins of major importance in cell division and morphology. Karyopherin subunit alpha 7 (*KPNA7*) is involved in nuclear protein transport (Tejomurtula et al., 2009), and *Kpna7*-deficient mice fail to develop to the blastocyst stage or show developmental delays (Hu et al., 2010). Whether *ABCC6*, *ACTL8*, *NLRP4*, *NLRP13*, and *WEE2* are involved in early embryogenesis remains unknown. Additionally, XPA-binding protein 2 (*XAB2*), whose expression does not appear to be stage specific, was particularly high in Ferticult (log2FC>5.5). *XAB2* plays a role in DNA repair (Hou et al., 2016) and is required for embryo viability (Yonemasu et al., 2005; Yanez et al., 2016). The activation of DNA repair mechanisms may be reflective of stress conditions experienced by pre-implantation embryos. Because embryos were cultured in the same medium after day 2 in our study and because the embryo is transcriptionally silent until EGA, we can hypothesize that the blastocyst transcriptome was influenced by alterations that occurred pre-compaction.

Finally, we investigated whether adding methionine to the culture medium, an essential amino acid whose concentration varies greatly between commercial media (Tarahomi et al., 2019), could affect embryo gene expression. Methionine is a precursor of S-adenosylmethionine, a key component in the one-carbon metabolism and methylation processes (Steegers-Theunissen et al., 2013). Methionine is necessary for proper embryo development, but in excessive concentration, it could negatively affect embryo abilities, as demonstrated in several animal models (Dunlevy et al., 2006; Rees et al., 2006; Kwong et al., 2010). Reassuringly, we did not identify any DEGs in sibling embryos cultured in Global until day 5, with or without methionine supplementation (concentration nearly thrice that in the Global medium), suggesting that excessive methionine concentration from day 2 did not have a major influence on the blastocyst’s transcriptome. It is possible that the original methionine concentration in the Global medium (50 μM as evaluated in Morbeck et al. (2014)) and the supplementation concentration (200 μM) assessed in this study are both within the physiological range. Consequently, the absence of significant differences after supplementation would be normal. Analyzing the early effects of methionine addition before EGA could be important.

Evidence that the embryo culture medium can impact gene expression has long been described in animals (Rinaudo and Schultz, 2004; Schwarzer et al., 2012; Feuer et al., 2017). Interestingly, in pig, adding reproductive fluids during *in vitro* culture allows producing blastocysts with closer chromatin and transcriptomic profiles compared to natural conditions (Canovas et al., 2017). It will be important to develop culture media closer to natural fluid even if we showed that the embryo is highly adaptable to different conditions. Additionally, if this study is reassuring, we might not forget that many other processes in the IVF laboratory environment constitute environmental stressors (temperature, pH, co-culture, light, oxygen tension, and manipulation). In our design, culture conditions other than culture media were identical for all samples, but a gamete or an embryo exposed to a stressful condition might be even more vulnerable to other stressors. In addition, identical freezing protocol was used, the slow freezing protocol, now optimized by vitrification. This freezing protocol might be a factor of the cumulative stress effect.

We cannot rule out that some differential expression observed at the blastocyst stage did stem from differences in embryo morphology between the groups, even if the blastocysts included were mostly B2. However, it is likely that if there are morphological differences, they may be substantially related to the use of the different culture media. In this study, we cannot exclude specific effects of couple characteristics (stimulation protocol, age, and infertility causes) on the embryonic transcriptome, but we showed that maternal age did not change the overall results.

For the first time in humans, we employed single-embryo RNA-seq on a unique collection of day-2 and -5 embryos to assess to what extent different culture conditions might affect the developing embryo transcriptome. Even though marked transcriptomic differences were observed between culture media at day 2, when embryos totally deprived in amino acids during their first days of development were returned to favorable culture conditions, these differences were reduced at the blastocyst stage. The few differences observed at day 5 may be attributed to a delay in molecular processes specific to the use of one medium. Altogether, our study emphasizes the abilities of the embryo to recover an expected transcriptomic landscape post-compaction. Consecutively, to rule out potential long-lasting epigenetic effects, it would be important to investigate whether the methylome also adapts to different media formulations. In addition, whether different embryo culture media used post-compaction could modulate the embryonic transcriptome, and notably, the expression of genes characteristic of lineage specification remains to be elucidated.

## Data Availability

The datasets presented in this study can be found in online repositories. The names of the repository/repositories and accession number(s) can be found below: GEO accession number: GSE212811.
