# Supplementary material for: Assessing the influence of distinct culture media on human pre-implantation development using single-embryo transcriptomics
Source: Front Cell Dev Biol. 2023 Jun 26;11:1155634. doi: 10.3389/fcell.2023.1155634 (PMC10330962; doi:10.3389/fcell.2023.1155634)
Supplement: Supplementary file 8 [file DataSheet1.DOCX]

Supplementary Material

Assessing the influence of distinct culture media on human pre-implantation development using single-embryo transcriptomics

Bastien Ducreux^1^, Julie Barberet^1,2^, Magali Guilleman^1,2^, Raquel Pérez-Palacios^3^, Aurélie Teissandier^4^, Déborah Bourc’his^4^, Patricia Fauque^1,2*^

*** Correspondence:** Pr Patricia Fauque : patricia.fauque@chu-dijon.fr

# Supplementary Methods

Freezing/thawing procedure

The freezing procedure was performed following a slow freeze protocol (2°C/min from 20°C to −7°C, manual seeding, 0.3°C/min to −30°C, and 35°C/min from −30 to −150°C). An adaptation of a freezing thawing procedure (FREEZE-KIT 1™ and THAW KIT 1™, Vitrolife, Göteborg, Sweden) was performed. Briefly, the straws were loaded successively with sucrose 0.2 M, air (1 cm), the embryo in 1.5 M propanediol + 0.1 M sucrose, air (1 cm) and 1.5 M propanediol + 0.1 M sucrose. At the time of thawing, the straws were kept at RT for 2 min, at 37°C for 3 min and at RT for 1 min and the straw contents were then mixed gently for 10 s. The embryo, in a solution of propanediol and sucrose, was then transferred to IVF medium and then immediately used for the single embryo RNA-seq.

# Supplementary Figures and Tables

## Supplementary Figures


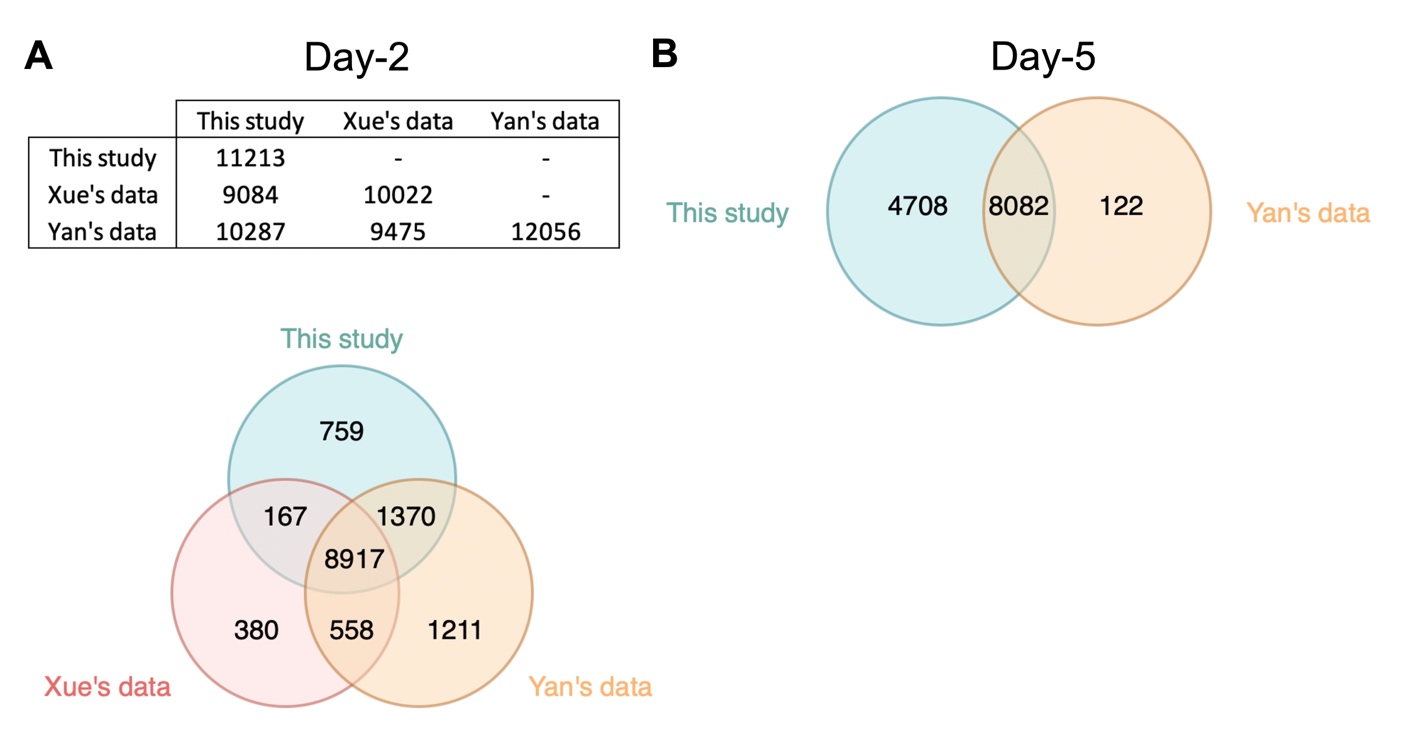


**Supplementary Figure S1.** Overlap of expressed genes in our study with Yan et al. and Xue et al. data. (**A**) Table and Venn diagram indicating the number of expressed genes that overlap between studies at day-2. (**B**) Venn diagram indicating the number of expressed genes that overlap between studies at day-5.

**
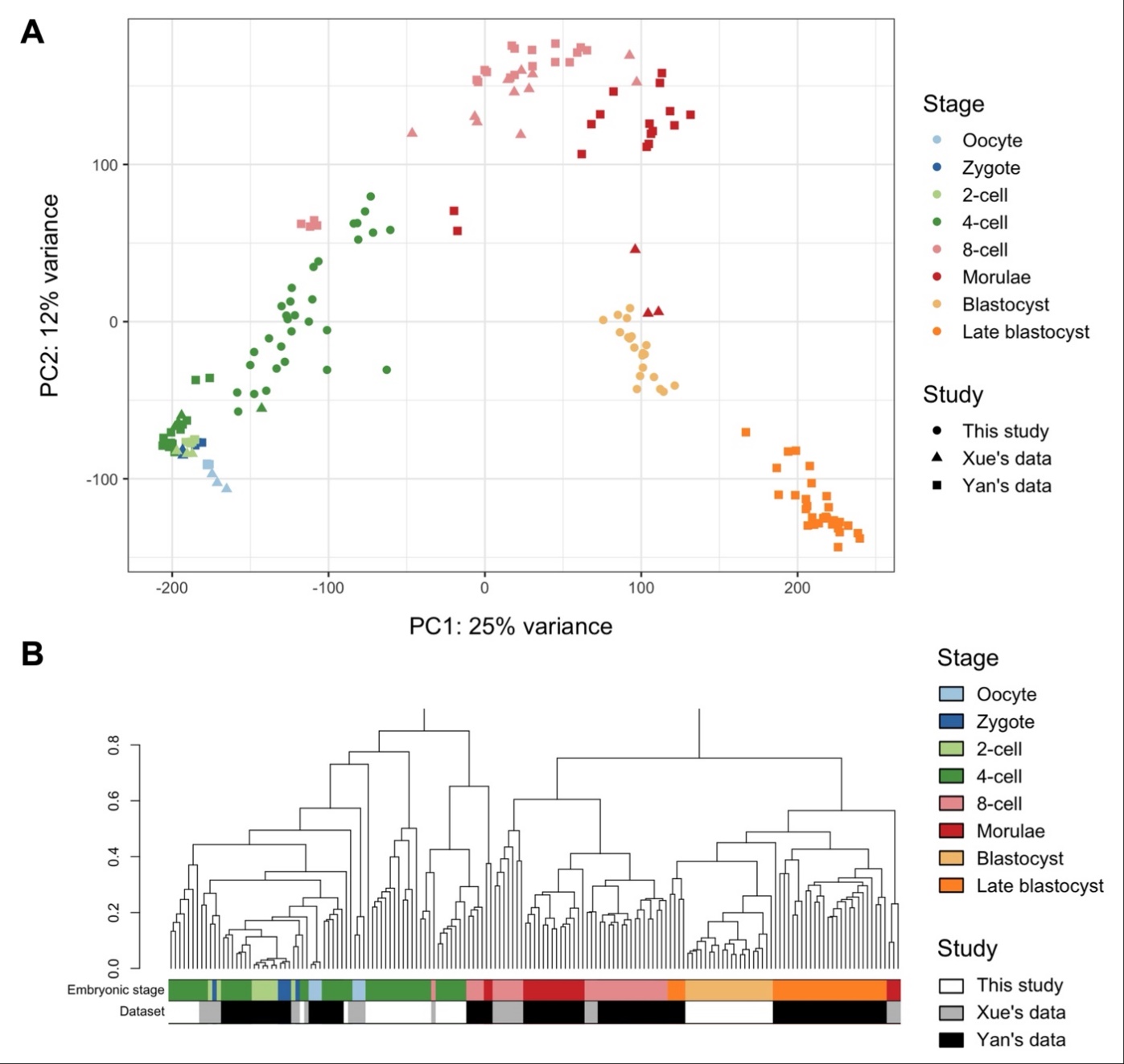
Supplementary Figure S2.** Comparison of global gene expression profiles between Yan et al., Xue et al. and this study’s datasets. (**A**) Principal component analysis of Yan et al., Xue et al. and this study’s datasets according to the normalized expression (log2(cpm+1)) of all expressed genes. Right panel displays PCA results for the embryos analyzed in this study only. (**B**) Hierarchical clustering of Yan et al., Xue et al. and this study’s datasets according to the normalized expression (log2(cpm+1)) of all expressed genes.

**
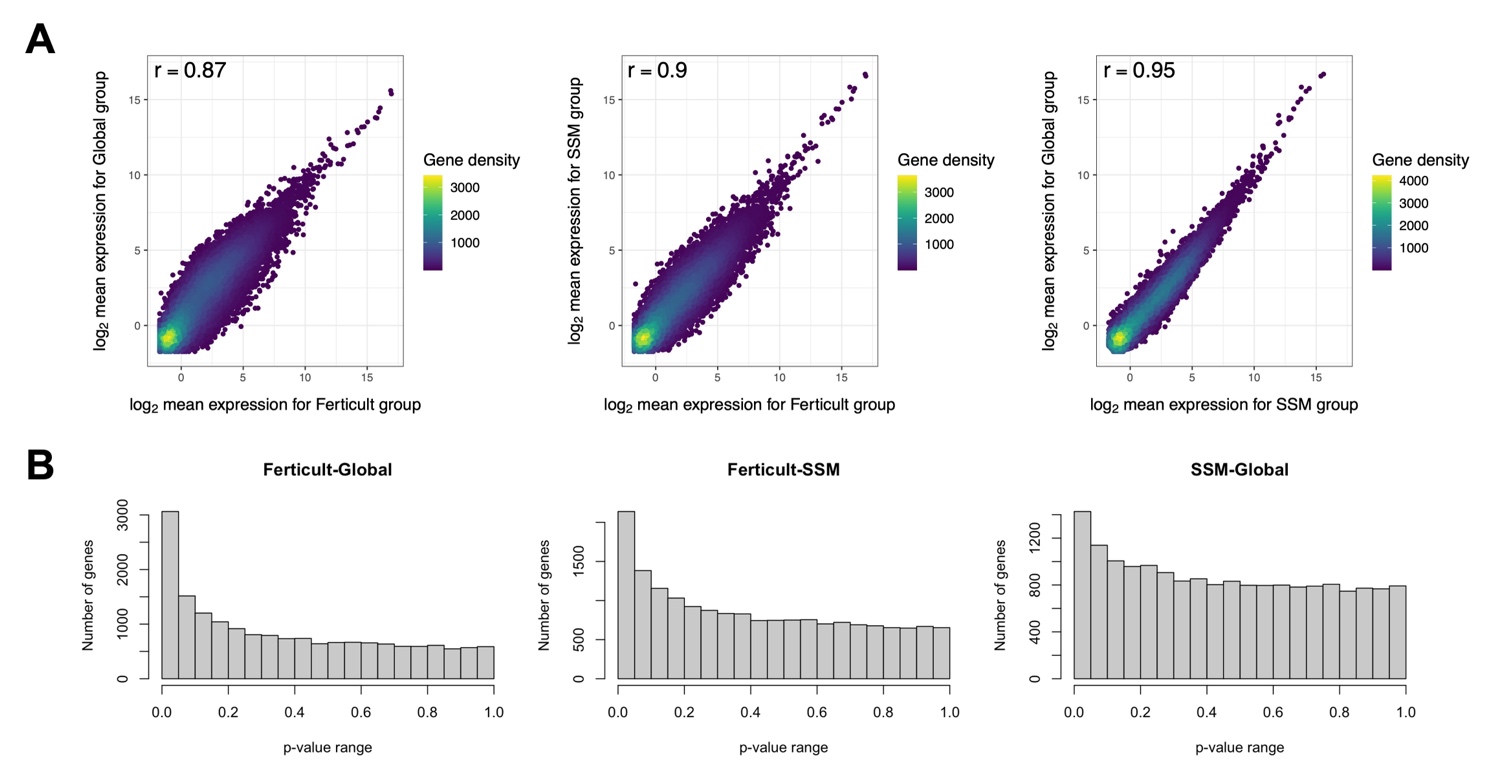
Supplementary Figure S3.** Statistics of the differential expression analysis for all three group comparisons. (**A**) Pairwise comparison of the levels of expression of all genes expressed between Ferticult, Global and SSM groups. R: Spearman’s correlation coefficient. Each point represents a gene. (**B**) Histogram of raw p-values in the three group comparisons.


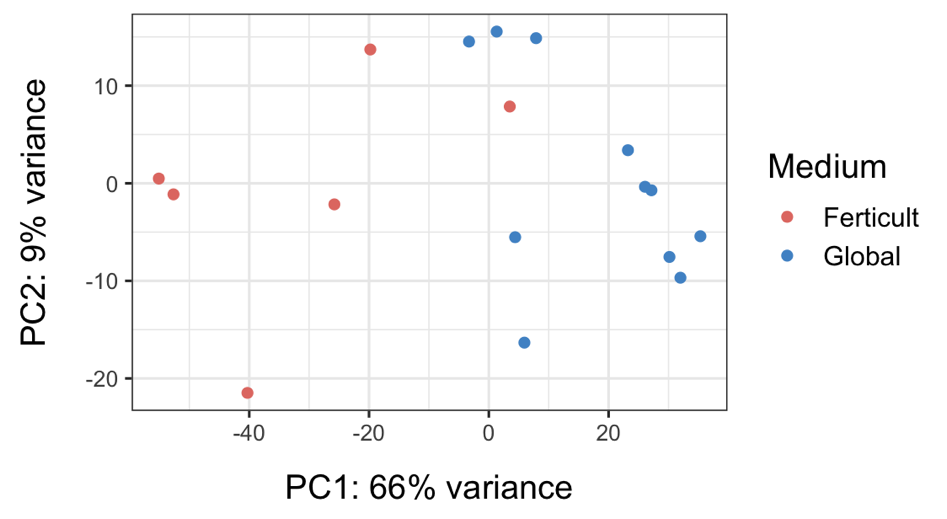


**Supplementary Figure S4.** PCA of Ferticult and Global samples at day-2 on the 266 DEGs.


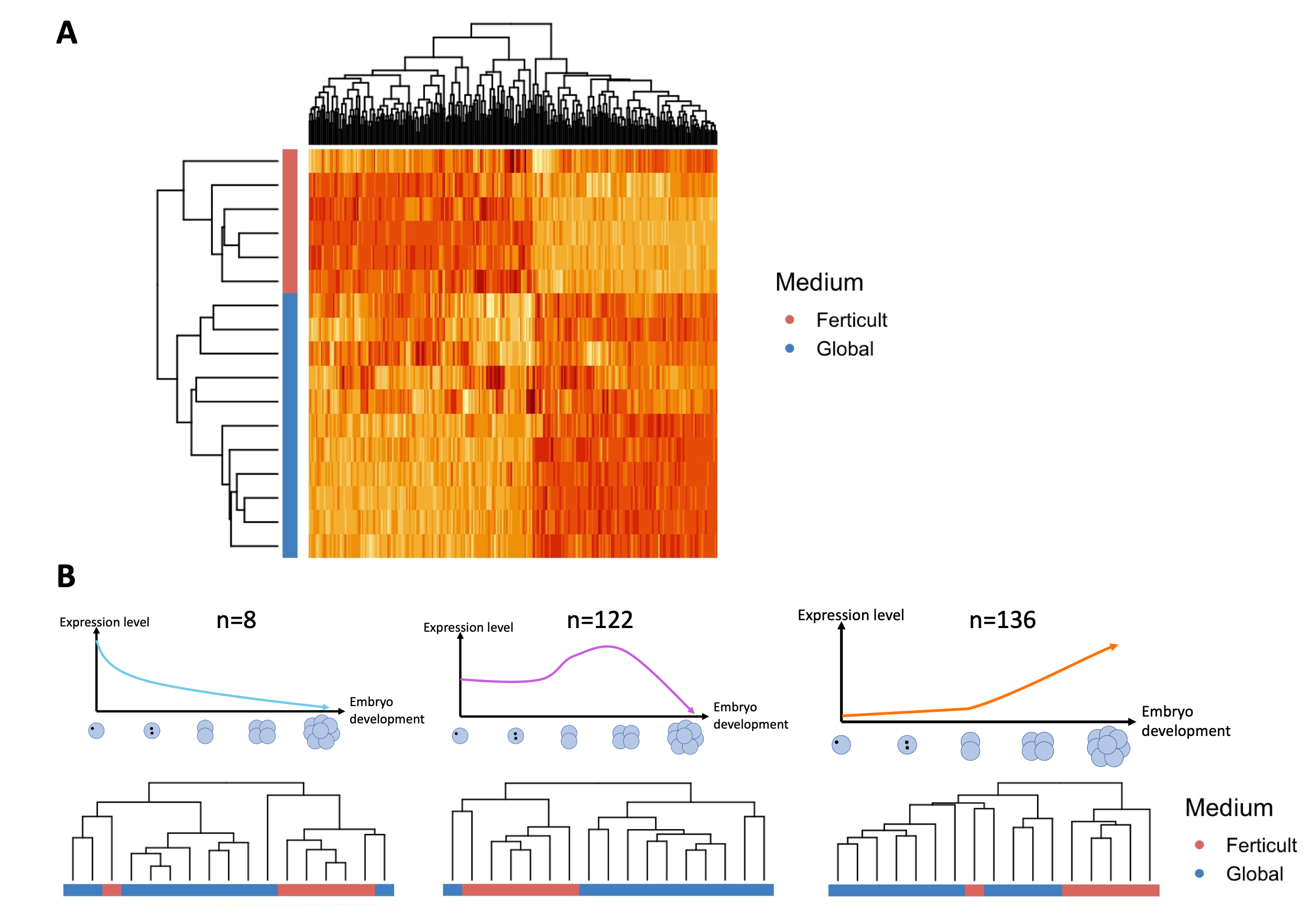


**Supplementary Figure S5.** Visualization of the 266 DEGs at day-2 in the Ferticult-to-Global comparison. (**A**) Heatmap according to their log2(cpm+1) expression. Samples are represented in rows and genes in columns. (**B**) Repartition of DEGs into strictly maternal, maternal and embryonic, and embryonic transcripts (from left to right) according to their pattern of expression along the first embryonic states assessed with Yan et al. (2013) data. Dendrograms represent the hierarchical clustering of genes per category, colored by culture medium groups.

**
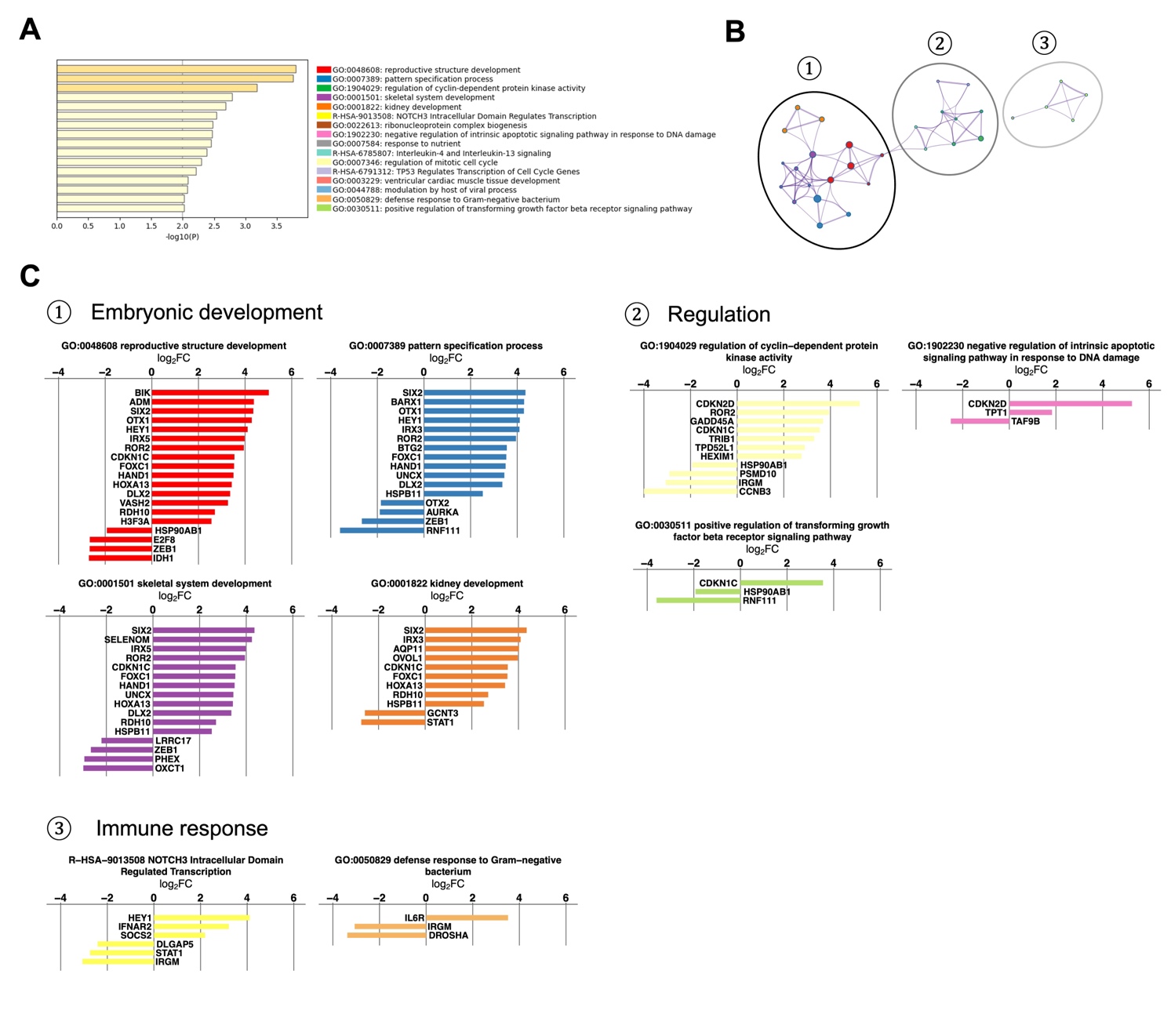
Supplementary Figure S6.** Detailed analysis of significant GO terms significantly over-represented in DEGs between Ferticult and Global at day-2. (**A**) Overview of most significant GO terms from clusters of significant pathways over-represented in day-2 differentially expressed genes (DEGs). (**B**) Network of all GO significantly enriched terms, colored by their representative GO terms. Three clusters of GO terms interaction were identified. Each node is an individual GO term and its size indicates the number of genes included in the GO term. Thick links connect GO terms of high similarity. (**C**) Detailed expression of DEGs between Ferticult and Global at day-2 involved in clusters of enriched pathways. Barplot represents the expression level (log2(cpm+1)) for each gene in the cluster.
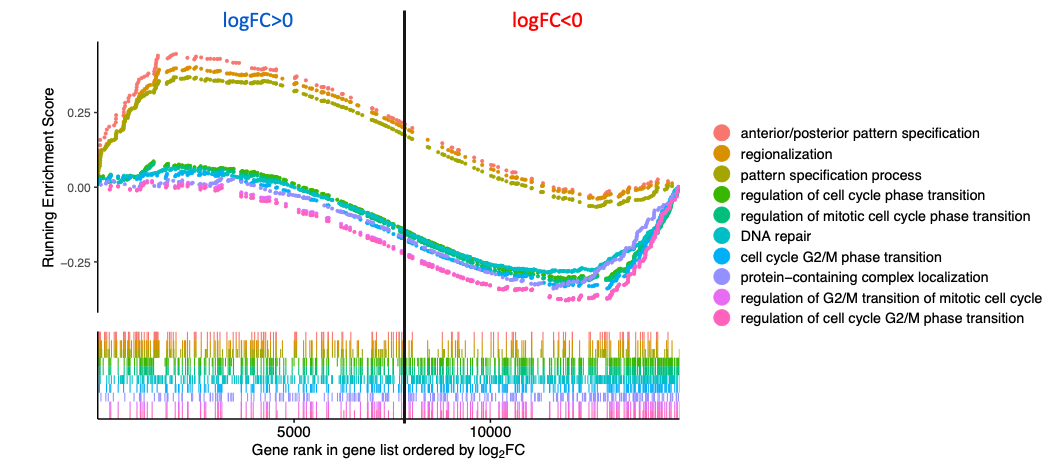
**Supplementary Figure S7.** Gene Set Enrichment Analysis (GSEA) of global gene expression changes in Ferticult-to-Global comparison. Only the top-10 biological processes are shown. The enrichment score, represented by the curves, reflects the degree to which genes belonging to a specific GO (each gene is represented by a point in the curve) are over-represented in the first or last position of the overall gene ranking (genes ranked from the highest positive logFC to the highest negative logFC). Walking down the ranked list of genes, GSEA increases the enrichment score when a gene is encountered in the GO considered (bars at the bottom of the plot) and decreases the score if not (absence of bars at the bottom of the plot). A positive peak enrichment score indicates that genes belonging to the GO considered are over-expressed with Ferticult. The vertical line separates genes with positive logFC (left side) from those with negative logFC (right side) with Ferticult compared to Global. For example, concerning the genes involved in the anterior/posterior pattern specification pathway, we observe high density of genes with the largest positive logFC (pink curve), symbolized by a positive peak enrichment score. This pathway appears up-regulated with Ferticult.

**
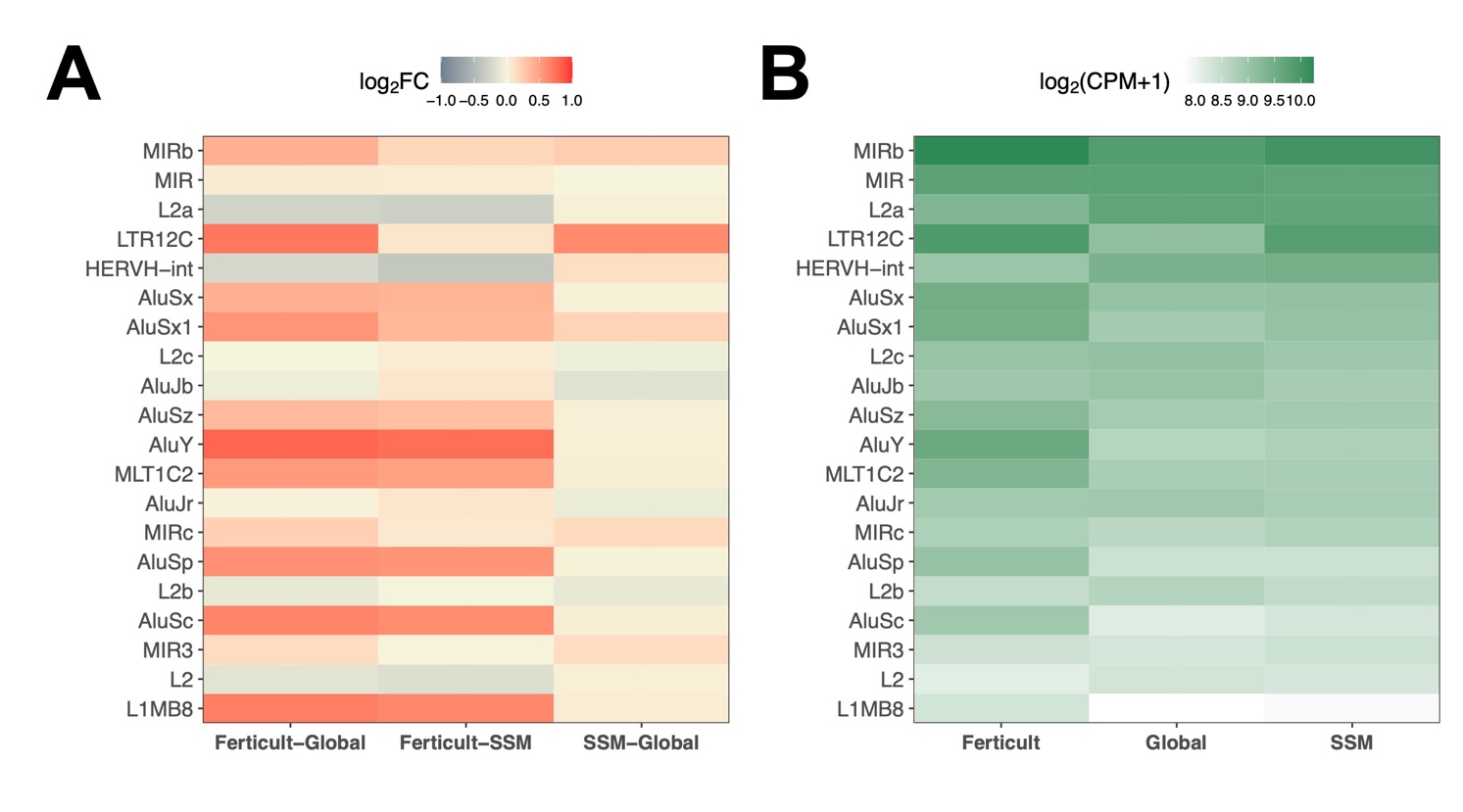
Supplementary Figure S8.** Differential expression analysis at day-2 focused on top-20 expressed transposable elements for all groups. (**A**) Heatmap of log2 fold change of transposable elements in all comparisons between culture media. (**B**) Heatmap of mean expression of transposable elements in all culture media groups. Log2 mean expression was calculated by taking the average log2(cpm+1) expression.

**
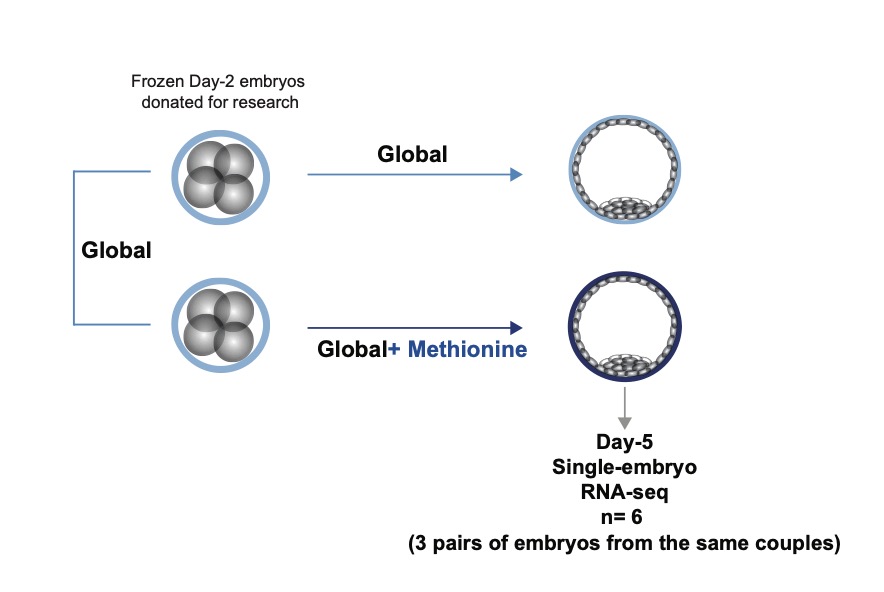
Supplementary Figure S9.** Study design of the experiment regarding methionine supplementation in the culture medium at day-2.

**
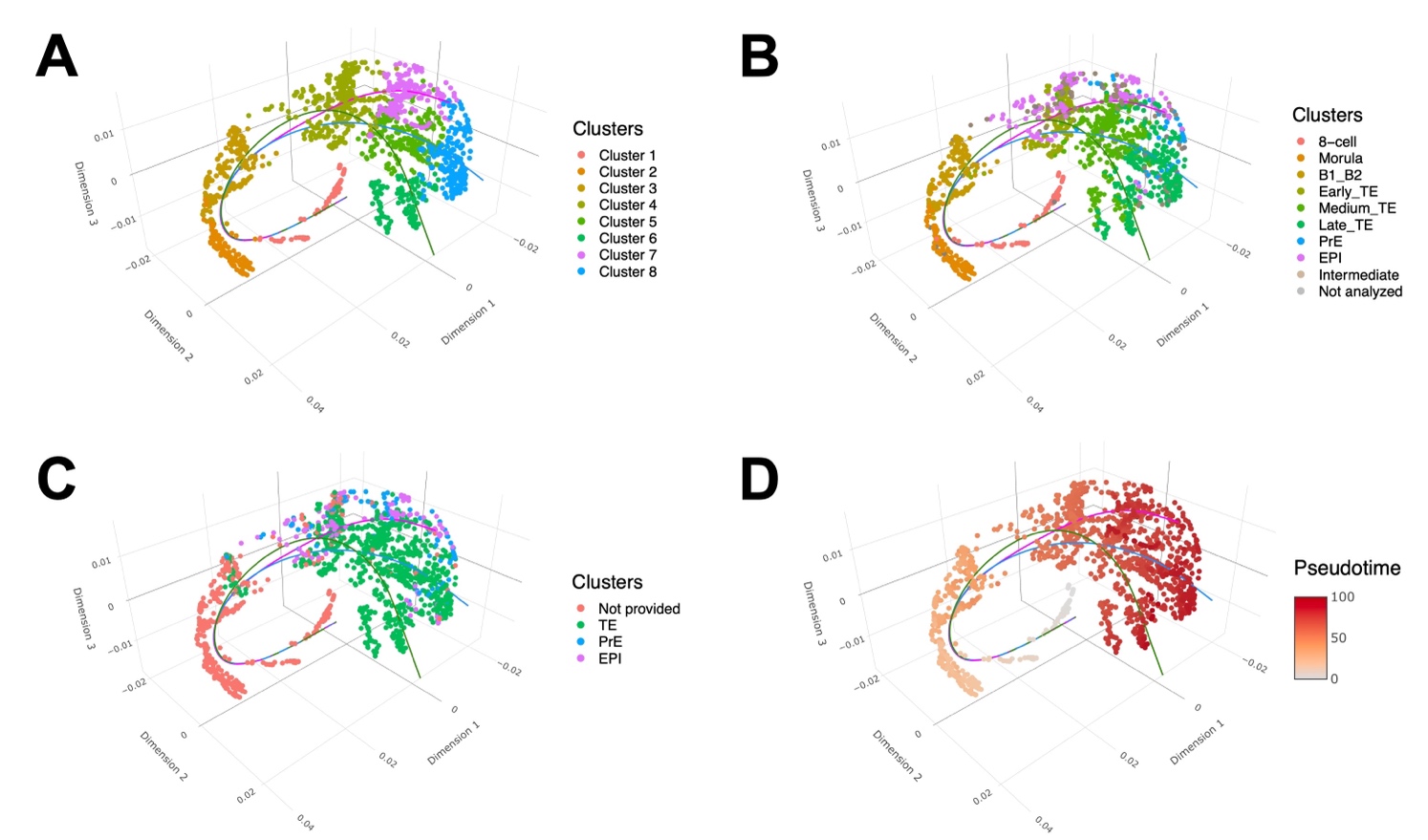
Supplementary Figure S10.** PHATE reduction of Petropoulos’ (2016) embryo scRNA-seq data. Each point represents one of the 1529 cells in the dataset. Curves indicate the trajectory of the three inferred lineages by slingshot. (**A**) Colored by k-means clusters identified in this study. Cells were grouped into 8 clusters according to their global expression of expressed genes. This clustering was used as input in slingshot to depict the global structure of underlying embryonic lineages. (**B**) Colored by Meistermann et al. lineage inference. Authors applied UMAP dimensionality reduction on module eigengenes identified with WGCNA (Langfelder and Horvath, 2008) to cluster cells according to their association with gene expression signatures specific to developmental stages and lineages. (**C**) Colored by Petropoulos et al. original lineage inference. Authors used PCA dimensionality reduction to infer embryonic lineages of cells after day-5. (**D**) Colored by pseudotime inferred with slingshot in this study (arbitrary unit). Pseudotime of the second lineage has been chosen for the purpose of visualization. Earliest embryonic stages in the dataset are gray colored, while dark red colored points are indicative of the latest cells timepoints.
